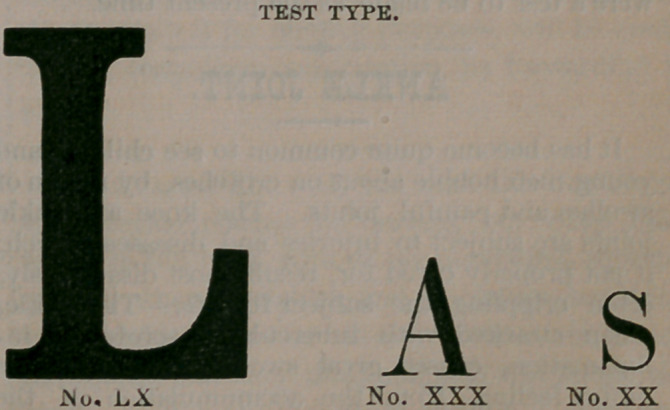# Cataract

**Published:** 1875-10

**Authors:** 


### The Bistoury.

ELMIRA, N. Y., OCT, 1875.

The BISTOURY is published Quarterly, upon the 1st of April, July, October and January, at Fifty Cents a year, in advance.

### CATARACT.

This word is derived from the Greek, signifying "to tumble down,"—hence, a deprivation of sight, which comes on as if a veil had fallen before the eyes.

Familiar as has this term become, yet, astonishingly few are the persons, even among the refined and intellectual classes of community, who have a correct understanding as to its real signification. More strange still is the circumstance that very few medical men, engaged in the general practice of medicine, that do not commit egregious blunders in their attempts at recognizing this obstruction to vision. Opacities of the cornea are often mistaken, by the careless observer, for cataract, and are usually so designated by laymen. If the reader will recall the fact that the clear, transparent portion of the eye, in front, is called the *cornea*, and will refer to what we have heretofore written of the diseases of this portion of the eye, it will be seen that this hard, clear, coating, from its prominent and exposed position, is liable to wounds and consequent inflammation, apt to be followed by ulceration, in the healing of which, white or pearl-colored scars or opaque spots are left, which obstruct vision in proportion to their size, location and density. These opacities are often caused, too, through the application of patent eye-waters to inflamed eyes, when, should there chance to exist a small abrasion of the cornea, a deposit of lead will take place, (as all patent "eye-waters" contain sugar of lead,) which leaves a yellowish-white spot, indelibly affixed, and when located immediately over the pupil, bears somewhat of a resemblance to cataract, to one unfamiliar with eye diseases. These scars are generally named by the common people "films," "pearls," or "skins," which they suppose can be removed surgically, by the oculist; but there exist very few opacities of the cornea that can be obliterated by any means, although we have known instances where so-called doctors, (of lamentably slender qualifications,) have attempted to remove with a knife, these "films,"—which they designated "cataracks,"—only to entail upon the patient untold suffering, and the destruction of his eyes.

In order that our readers may distinguish readily between an opacity of the cornea and cataract, we here give a very excellent representation of a central scar upon the cornea:

(Fig. I.)

In this illustration the scar appears nearly round and almost entirely conceals the pupil, a small margin only appearing just below. From such an eye, a person would see indifferently, but could have the pupil enlarged, by cutting out a portion of the *iris*, below the opaque spot, in a manner heretofore described, so as to afford excellent vision. It will be observed that the opaque spot is upon the surface of the cornea. Should it be very dense and white, by rubbing the finger over the closed eye-lid, it will be found to project somewhat beyond the natural surface of the cornea. Observe, also, that the spot is not confined within the limits of the pupil, but may extend to one side or the other, or indeed, completely cover the cornea. Contrast this state of affairs with the illustration below, where we have a real cataract, characterized by a perfectly round, white spot, the exact size of the pupil, leaving the *cornea* entirely

(Fig. II.)

free from blemish. Place your hand over the patient's eye, so as to exclude the light, then remove it in a few moments, and you will discover that the pupil has enlarged,—*and so has the white spot!* Better still, place a drop of tincture of belladonna in the eye,—it will do no harm,—keep your patient from the light for a half hour, then inspect his eye. You will find the pupil to be largely dilated, and that the cataract is proportionately enlarged, corresponding precisely to the size of the pupil. It will also be found that your patient will see more light by reason of this dilatation; a circumstance seized by the traveling quack doctor, to induce a cataract patient to believe that his vision can be restored by the aid of medicines and without the necessity for calling in the surgeon for an operation. He will widely dilate the pupil with a solution of atropia, enabling the patient, sometimes, where the lens has not become very opaque, to see well enough to walk alone. In this manner, the confidence of the patient is obtained until a handsome fee is paid, when the itinerant doctor decamps as mysterously as he came. Many advertising charlatans announce, "cataracts removed without the knife." Their professions are as false as are their practices dishonest. No medicine, either locally or constitutionally, can be of any possible avail in restoring vision lost from cataract. This fact will appear more forcibly when we shall have explained what cataract is. In order to accomplish this readily, we must introduce another cut and give you a short lesson on the anatomy of the eye:

(Fig. III).

This represents the eye, cut in two, directly through its centre, while we are looking at it sidewise. At *a b* are represented the eye-lids closed over the eye. *d.* is the *cornea*, the clear, front part of the eye, before referred to. *f* is the pupil, being simply a round hole through the *iris*. The iris is a curtain, hanging before the lens, and gives color to the eye—being blue, hazel, gray, etc., its lower border reaching to *k*. Next, we reach the lens,—the oval body marked *h*—and now we have arrived at the location of our cataract.

The object of this lens, is to refract the rays of light coming from any given object, and to concentrate them upon the retina, (represented at 3 and 5), where the image of the object viewed is depicted. In order for the eye to accomplish this satisfactorily, it becomes absolutely necessary that the lens should be perfectly clear and transparent, which it is in health, resembling the purest crystal. Now, in cataract, precisely the reverse occurs,—the lens, through lack of the proper conditions to keep it clear and healthful, becomes gradually hazy, nebulous, opaque, until no light can pass through it. What then occurs? Look at our cut and see! Do you observe how much larger the diameter of the lens is than the hole we call the *pupil*? Do you see that it more than fills the pupil *f*? This being the case, when the lens becomes *white* or opaque, all light is completely excluded from the *retina*, 3 5, where the seat of vision exists. This accounts, too, for the cataract appearing larger when you dilate the pupil. The lens being *behind* the pupil, and so much larger, of course, more of it is brought to view by dilating it. Does it now appear plain to you why the cataract should be *in* the pupil, perfectly round, and varying in size with the pupil, resembling Fig. II? We think you must have a fair understanding as to the locality of a cataract, and see, too, how impossible it would be to remove such a substance from within the eye, by any sort of medication, and how important it must be to secure the very best skill that can be had to perform the operation that we are about to describe.

Several different methods of operating for cataract are practiced by ophthalmic surgeons at this day, all possessing more or less merit, and all resulting, as a general rule, successfully. The old plan, of pushing the cataract out of the way of the pupil, with a needle, called "depression of cataract," is rarely practised now-a-days. It consisted in thrusting a delicate, flat pointed needle through the *sclerotic*, (white of the eye), near the cornea, and carrying it behind the iris and pupil, until it reached the lens. (Fig. IV.)

The lens was then carefully and steadily pushed down, behind the iris, until it lay near the bottom of the eye and out of the way of the passage of the light to the nervous portion, or retina.

While this operation is quite easy to accomplish, and often affords excellent vision, yet, the lens is liable to leave its bed and pop up into its old position to obscure the pupil, producing the so-called secondary cataract, or to excite inflammation by coming in contact with the internal membranes of the eye, thus destroying vision. It is now never practised by skillful oculists, save upon very young children, when the needle is used for breaking up the lens, leaving the fragments for absorbtion, and necessitating several operations before the eye is made clear.

(Fig. IV.)

The position of the lens will be best understood by the little figure below, where B represents the natural position of the lens, the dotted line the direction of its depression, and B the position where the needle left it.

(Fig. V.)

But *the* operation, requiring a clear head and steady hand, and when neatly done, giving most glorious results, is the one performed for removing the lens entire from the eye. This is perhaps the most delicate operation in surgery, and is wholly in the hands of specialists, very few general surgeons in our largest hospitals even, being willing to attempt it. The operation is called the extraction of cataract, and consists in making an opening in the cornea, with a very delicate knife, of sufficient size to allow of the exit of the lens. This opening is made either at the lower margin of the cornea, called the lower section, or at the upper margin, then named the upper section. The latter is oftenest practised and is the most difficult to accomplish, but usually gives the best results, as the upper lid best covers and protects the wound, ensuring a more speedy union than when the lower section is made. Oculists have their pet hobbies with reference to making this cut. Some follow near the margin of the cornea, cutting it nearly half in two, when it is named lower or upper "flap extraction." Others thrust the knife just within the white margin, near the cornea, making a straight line of the wound, and call it "Von Graefe's Modified Linear Extraction," after the celebrated German oculist who first adopted this plan. Numerous other plans for making the incision exist, but all have the same object in view.

Like all operators, we have *our* particular notion as to how the operation should be done, and the plan we adopt is substantially as follows: We differ with all other operators of whom we have knowledge, in the matter of giving chloroform or ether, or in holding the eye with fixation forceps.

We do neither, and have never had any difficulty in extracting a lens without holding the eye with forceps, consequently no anæsthetic becomes necessary. By this plan, we are rid of the nausea produced by the chloroform,—a very important matter in the success of an operation for cataract, and of the pain and inflammation that follows the use of the forceps that holds the eye quiet. Out of nearly four hundred operations for cataract, we have only found it necessary in one or two instances, to administer chloroform, and then only by reason of extreme fright and "nervousness" of the subjects. We think that every individual, without an exception, in the table of our last fifty operations, here given, will testify that the pain of the operation was so trifling as not to be worth mentioning.

(Fig. VI.)

The patient is placed upon his back, on a lounge, before a window, a speculum for holding the lids apart, placed in position, and is directed to look down and to use all his effort in keeping his eye rolled in that direction. While we are talking with him about the different steps of the operation, our knife is thrust through the cornea, and the point pushed on until it emerges at the opposite side, as beautifully portrayed in the above illustration.

We then carefully draw the knife back and forth, with a sort of sawing motion, following the rim of the cornea, if it is a "flap extraction," cutting deeper into the sclerotic, if it is a "modified linear extraction," until the knife is brought out at the top of the wound, as seen in the cut. Strange to say, all this is accomplished without pain, for the cut is made through tissue as devoid of nerves of sensation as your finger nail. Next, a little hook or a delicate forceps is thrust through the wound and grasps the iris at the margin of the pupil, and drawing it through the wound, a piece of it is snipped off with the scissors, leaving the pupil to appear in this shape:

(Fig. VII.)

The object in making this opening through the *iris* is to sufficiently enlarge the pupil to enable the lens to pass, which we have seen is much larger than the pupil. The slit is hid under the upper lid, and is not observed. This accomplished, the next process is to lacerate the capsule or membrane that holds the lens in position, but this is not always necessary, as the lens generally ruptures its own capsule, and floats forward toward the wound as soon as the cut is made. The patient is now cautioned to look downward, with all his might, while two delicate rubber spatulæ are pressed, one against the lower portion of the eye, the other upon the upper lip of the wound, when the lens is seen to float forward, and in a twinkling pops out upon the face, leaving the pupil as clear as crystal. Immediately the window is shaded, and the patient counts his fingers, sees your face, can distinguish your nose and eyes, and says the light is very bright and altogether lovely. The eye is now carefully packed with lint, over the lids, and a flannel roller bandage applied in such a manner as to prevent the eye from moving, and to keep the lips of the wound in perfect apposition. The patient is now undressed and placed in bed, where he is left for two or three days, and within a week, the bandage is removed and the eye permitted to encounter the light of a room with blinds closed. Day by day, more light is admitted, until from two to three weeks have passed, when "cataract glasses" are fitted to his eye,—one to see to walk with, another to read, and he is then allowed to go home.

We have been thus minute in giving every particular about the operation in order to answer questions that are constantly asked us by patients from a distance, hoping to make it so plain as to relieve us from much of the letter-writing to which we are now subjected.

We now give a list of the last fifty operations for cataract that have occurred in our Institution, showing a success that fills us with pride, not only for the perfection to which this operation has been brought, but also for the extreme satisfaction it has afforded us in bringing these blind people to the blessings of vision, and so making them our true, grateful and loving friends. As we look over the list, we recall the faces of every one of them, and remember the eager satisfaction with which they inspected our face for the first time, and the many merry jokes that were cracked at our expense. We hope they will pardon the publicity given to their names, when they consider it may be the means of bringing many others from the darkness which once envailed them, to the beautiful bright, sunlight, which they now enjoy.

In the accompanying report we give the resulting success, indicating the acuteness of vision by the size of the type able to be read, by means of cataract glasses of from 5 to 2½ inch focus. When a patient is able to distinguish No. 60 type at fifteen feet, we consider the case a success. It must be borne in mind that many of the cases here enumerated, were tested only within three or four weeks after the operation, at a time when the eye had not gained its full strength and power. Doubtless many who are noted as being able to read No. 60, could in two or three months afterward read as low as number VI. The figures refer to power of lens used.

TEST TYPE.

| L | A | S |
|---|---|---|
| No. LX | No. XXX | No. XX |

The type with which this journal is printed is No. VI.

This type is designated No. 1. and the individual who can read it after an operation for cataract, must possess excellent vision.

## Figures and Tables

**(Fig. I.) f1:**
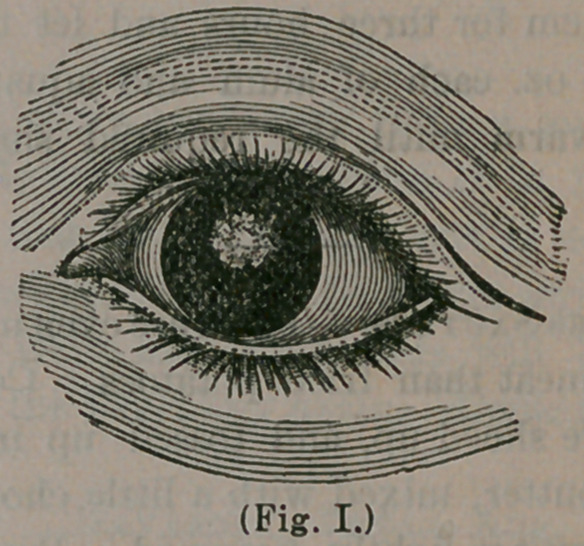


**(Fig. II.) f2:**
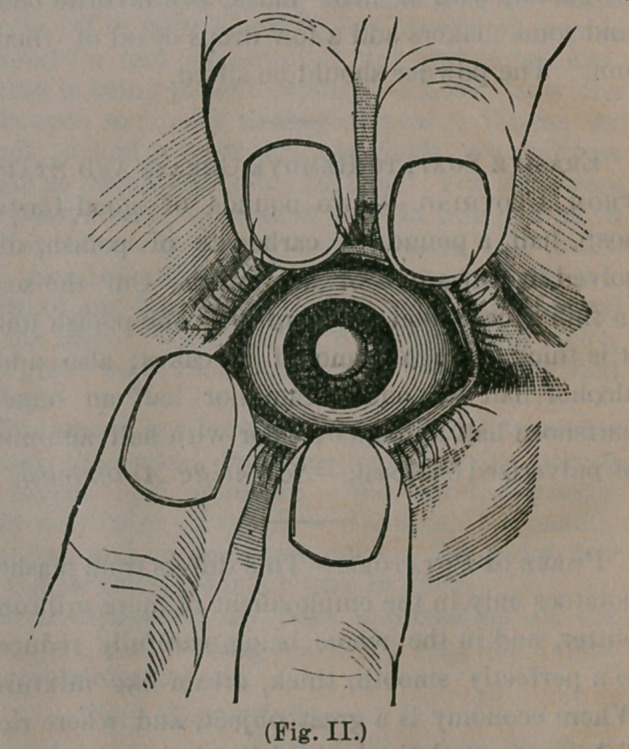


**(Fig. III). f3:**
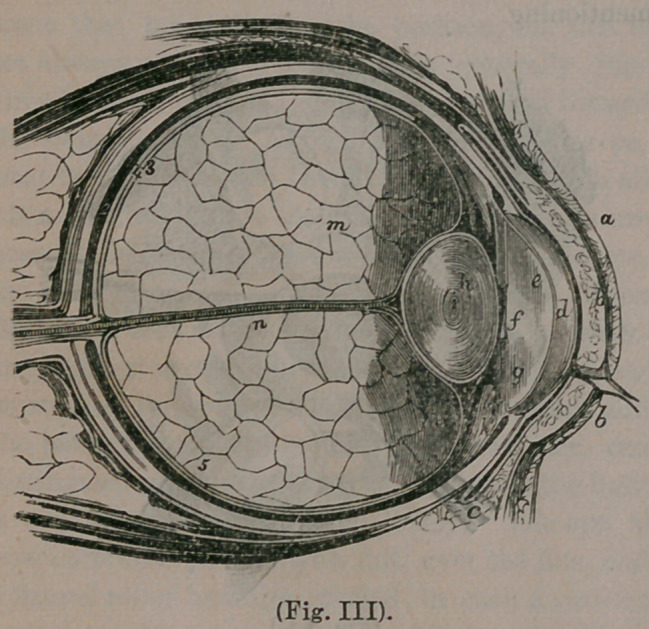


**(Fig. IV.) f4:**
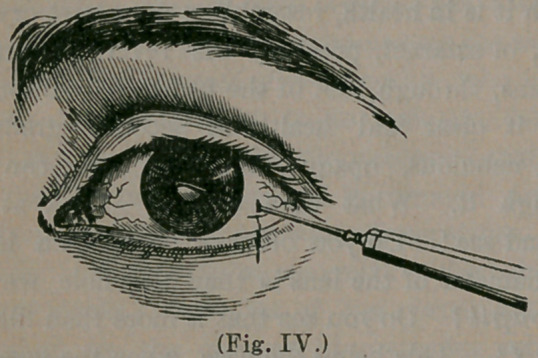


**(Fig. V.) f5:**
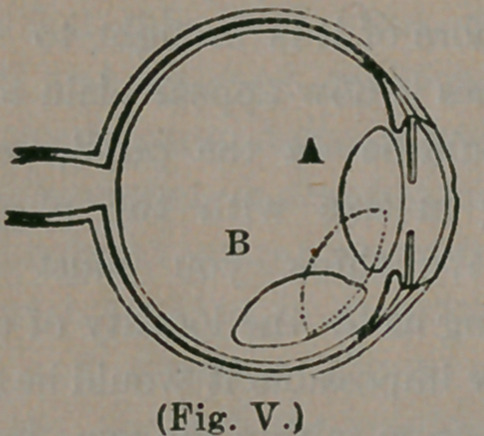


**(Fig. VI.) f6:**
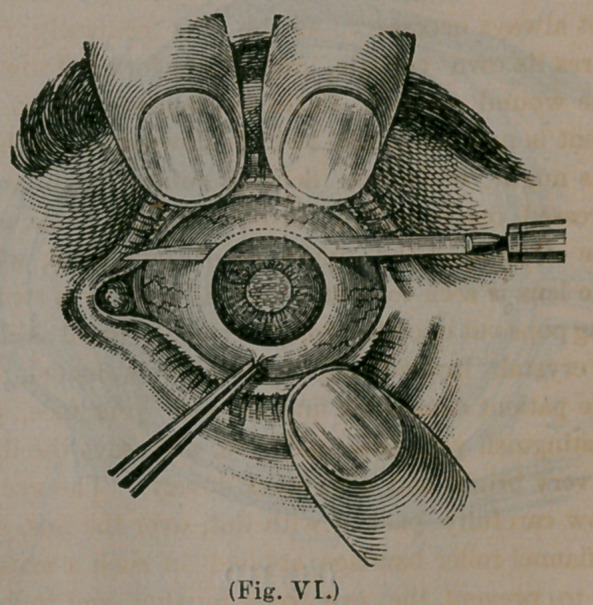


**(Fig. VII.) f7:**
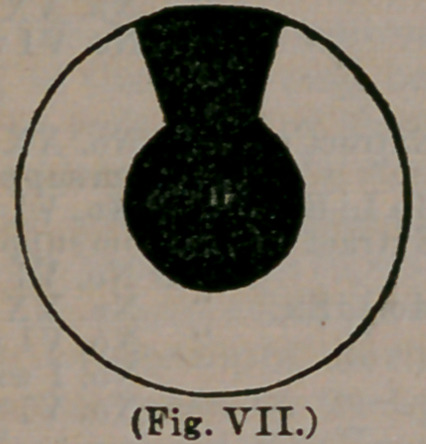


**Figure f8:**